# Supplementary material for: Meta-Analysis and Gene Set Enrichment Relative to ER Status Reveal Elevated Activity of MYC and E2F in the “Basal” Breast Cancer Subgroup
Source: PLoS One. 2009 Mar 9;4(3):e4710. doi: 10.1371/journal.pone.0004710 (PMC2650420; doi:10.1371/journal.pone.0004710)
Supplement: Dataset S1 — GSEA results from first screen. The file contains a zipped archive of the HTML results of the second GSEA screen. Reports for enriched or depleted datasets may be accessed from the “index.html” link within the my_analysis.GseaPreranked.1219975950625.rpt.zip ZIP archive. Enrichment plots are available for each of the 50 most enriched and depleted gene sets. (13.32 MB ZIP) [file pone.0004710.s001.zip › my_analysis.GseaPreranked.1219975950625.rpt.zip!//CHR14Q12.html]

Details for gene set CHR14Q12[GSEA]

|  || Dataset | zSco.ERneg.to.ERpos\_collapsed |
| Phenotype | NoPhenotypeAvailable |
| Upregulated in class | na\_neg |
| GeneSet | CHR14Q12 |
| Enrichment Score (ES) | -0.585001 |
| Normalized Enrichment Score (NES) | -1.7194922 |
| Nominal p-value | 0.015228426 |
| FDR q-value | 0.05550657 |
| FWER p-Value | 0.951 |
Table: GSEA Results Summary

  

Fig 1: Enrichment plot: CHR14Q12      
 Profile of the Running ES Score & Positions of GeneSet Members on the Rank Ordered List

  

| PROBE | GENE SYMBOL | GENE\_TITLE | RANK IN GENE LIST | RANK METRIC SCORE | RUNNING ES | CORE ENRICHMENT || 1 | BAZ1A |  |  | 2724 | 2.078 | -0.1303 | No |
| 2 | AP4S1 |  |  | 4992 | 1.159 | -0.2591 | No |
| 3 | AKAP6 |  |  | 6305 | 0.715 | -0.3321 | No |
| 4 | NPAS3 |  |  | 6322 | 0.710 | -0.3079 | No |
| 5 | EGLN3 |  |  | 6355 | 0.700 | -0.2852 | No |
| 6 | CTSG |  |  | 7080 | 0.456 | -0.3233 | No |
| 7 | CIDEB |  |  | 7439 | 0.332 | -0.3383 | No |
| 8 | PAX9 |  |  | 8864 | -0.239 | -0.4367 | No |
| 9 | OSGEP |  |  | 9608 | -0.623 | -0.4702 | No |
| 10 | SCFD1 |  |  | 11136 | -1.691 | -0.5244 | Yes |
| 11 | NOVA1 |  |  | 11652 | -2.168 | -0.4855 | Yes |
| 12 | C14ORF124 |  |  | 12577 | -3.385 | -0.4336 | Yes |
| 13 | PCK2 |  |  | 12617 | -3.462 | -0.3125 | Yes |
| 14 | FOXA1 |  |  | 13107 | -4.857 | -0.1753 | Yes |
| 15 | NUBPL |  |  | 13191 | -5.337 | 0.0097 | Yes |
Table: GSEA details [plain text format]

  

Fig 2: CHR14Q12: Random ES distribution      
 Gene set null distribution of ES for **CHR14Q12**
